# Increased Plasma Concentrations of Vitamin D Metabolites and Vitamin D Binding Protein in Women Using Hormonal Contraceptives: A Cross-Sectional Study

**DOI:** 10.3390/nu5093470

**Published:** 2013-09-05

**Authors:** Ulla K. Møller, Susanna við Streym, Lars T. Jensen, Leif Mosekilde, Inez Schoenmakers, Shailja Nigdikar, Lars Rejnmark

**Affiliations:** 1 Department of Endocrinology and Internal Medicine, THG, Aarhus University Hospital, Tage Hansens Gade 2, DK, Aarhus 8000, Denmark; E-Mails: susanna.vid.streym@gmail.com (S.S.); leif.mosekilde@gmail.com (L.M.); rejnmark@post6.tele.dk (L.R.); 2 Department of Clinical Physiology, Glostrup University Hospital, Copenhagen DK-2900, Denmark; E-Mail: lars.thorbjoern.jensen@regionh.dk; 3 MRC Human Nutrition Research, Cambridge CB1 9NL, UK; E-Mails: inez.schoenmakers@mrc-hnr.cam.ac.uk (I.S.); shailja.nigdikar@mrc-hnr.cam.ac.uk (S.N.)

**Keywords:** hormonal contraceptives, 25hydroxyvitamin D, 1,25-dihydroxyvitamin D, vitamin D binding protein, parathyroid hormone, calcitonin, bone turnover, bone mineral density

## Abstract

Use of hormonal contraceptives (HC) may influence total plasma concentrations of vitamin D metabolites. A likely cause is an increased synthesis of vitamin D binding protein (VDBP). Discrepant results are reported on whether the use of HC affects free concentrations of vitamin D metabolites. *Aim*: In a cross-sectional study, plasma concentrations of vitamin D metabolites, VDBP, and the calculated free vitamin D index in users and non-users of HC were compared and markers of calcium and bone metabolism investigated. *Results:* 75 Caucasian women aged 25–35 years were included during winter season. Compared with non-users (*n =* 23), users of HC (*n =* 52) had significantly higher plasma concentrations of 25-hydroxyvitamin D (25OHD) (median 84 interquartile range: [67-111] *vs.* 70 [47-83] nmol/L, *p =* 0.01), 1,25-dihydroxyvitamin D (1,25(OH)_2_D) (198 [163-241] *vs.* 158 [123-183] pmol/L, *p =* 0.01) and VDBP (358 [260-432] *vs.* 271 [179-302] µg/mL, *p <* 0.001). However, the calculated free indices (FI-25OHD and FI-1,25(OH)_2_D) were not significantly different between groups (*p >* 0.10). There were no significant differences in indices of calcium homeostasis (plasma concentrations of calcium, parathyroid hormone, and calcitonin, *p >* 0.21) or bone metabolism (plasma bone specific alkaline phosphatase, osteocalcin, and urinary NTX/creatinine ratio) between groups. *In conclusion:* Use of HC is associated with 13%–25% higher concentrations of total vitamin D metabolites and VDBP. This however is not reflected in indices of calcium or bone metabolism. Use of HC should be considered in the interpretation of plasma concentrations vitamin D metabolites.

## 1. Introduction

Vitamin D (calciferol) is obtained from endogenous synthesis in the skin in response to solar UV-B radiation and intake from the diet and supplements [1,2]. Once in the circulation, calciferol is converted to 25-hydroxyvitamin D (25OHD) in the liver and, subsequently, to its circulating biologically active form 1,25-dihydroxyvitamin D (1,25(OH)_2_D) in the kidney [3]. This conversion may also occur in other tissues for auto- or paracrine actions [4]. It has been estimated that 85% to 90% of 25OHD and 1,25(OH)_2_D is bound to vitamin D binding protein (VDBP) [5], 10% to 15% to albumin, whereas only a very small fraction (<0.1%) circulates in its free form [5,6]. VDBP binding protects vitamin D metabolites from hydroxylase-mediated catabolism, affects their cellular uptake, and modulates their biological activity [5,6].

Total plasma concentrations of 25OHD are considered an indicator of vitamin D status due to its long plasma half-life (approximately 15–35 days) and lack of hormonal control of the hepatic 25-hydroxylase [3].

Vitamin D is known to affect several health outcomes. Classically, low vitamin D concentrations are known to be associated with an increased risk of myopathy, rickets or osteomalacia, and low bone mineral density and fracture. In a number of recent studies, an impaired vitamin D status has also been associated with various adverse non-skeletal health outcomes such as an increased risk of malignancies or cardiovascular diseases [1]. 

Plasma 25OHD concentrations are influenced by many factors. In addition to variations in UVB-exposure and dietary intake, 25OHD concentrations are influenced by several host factors such as age, adiposity [2,7,8], ethnicity, and skin tone as well as certain genotypes [8,9], and plasma VDBP concentrations [5]. 

Pregnancy is known to be associated with an increase in VDBP through its oestrogen mediated increase in synthesis [10,11]. Plasma concentrations of 25OHD are reported to be unaltered and 1,25(OH)_2_D to be elevated compared to non-pregnant women [12,13,14]. The use of hormonal contraceptives (HC) may also affect 25OHD concentrations and metabolism due to their oestrogenic components. The limited data on the effects of HC on 25OHD concentrations report no change or an increase in total 25OHD [9,12,15,16,17], whereas most studies consistently report an increase in levels of 1,25(OH)_2_D and VDBP [10,15,18,19,20].

These data suggest that HC may cause differential effects on 25OHD and 1,25(OH)_2_D; the free 25OHD index (the molar ratio of 25OHD- to VDBP-concentrations) may be decreased due to an absence of a parallel increase in VDBP and 25OHD, whereas the free index of 1,25(OH)_2_D may remain unchanged.

In order to study the possible effects of HC, we compared plasma concentrations of 25OHD, 1,25(OH)_2_D, VDBP, and the calculated free vitamin D index in users and non-users of HC. In addition, we assessed possible impacts of HC on calcium homeostasis and bone metabolism.

## 2. Subjects and Methods

This paper reports a secondary analysis of the effects of HC on vitamin D metabolism in a subset of women participating in a population based controlled cohort study, using cross-sectional data obtained at baseline. The design of the study has previously been reported in detail [12,21]. In brief, we included 153 healthy Caucasian women, aged 25–35 years, trying to conceive, and 75 age-matched women not planning a pregnancy for the next 21 months. All women were recruited by direct mailing of 11,175 randomly selected women from a population of 21,317 women aged 25–35 years living in the community of Aarhus, Denmark. We obtained names and addresses from the Danish Civil Registration System. A total of 561 wished to participate, from which 333 were excluded as based on predefined exclusion criteria (Pregnant or breastfeeding at the start of the study (*n =* 85), known infertility (*n =* 46), miscarriage within last 6 months (*n =* 3), withdrawal or moved residence (*n =* 84), age, illness, foreign origin (*n =* 25), or responded after closure of recruitment (*n =* 90)). Analyses reported in this paper only include data obtained in the group of women (*n =* 75) not planning a pregnancy, of which 52 were using hormonal contraception (including oral, subdermal contraceptive implant, or hormonal spiral methods). They were all included between October 2006 and April 2007. The study was performed according to The Helsinki Declaration II. The study was notified to the Danish Data Protection Agency (#2004-41-4737) and approved by the Regional Scientific Ethical Committee of Aarhus County (#20040186).

### 2.1. Measurements

Standing height and body weight were measured (Seca, Sa-med, Kvistgaard, Denmark) wearing indoor clothing. Incident diseases and the use of drugs were recorded. Participants were asked to fill in a questionnaire on medical conditions, smoking habits, and dietary intake of calcium as well as use of calcium and vitamin D containing supplements. Dietary intake of calcium was assessed as previously described [22] and total calcium intake was calculated as dietary intake plus intake from supplements.

### 2.2. Biochemistry

A non-fasting blood sample was drawn between 8 a.m. and 2 p.m. according to standardized procedures and centrifuged at 4 °C with a relative centrifugal force of 2500 *g* for 10 min. Plasma was separated and stored at −80 °C until analyzed. Urine and plasma samples were assessed in batches, *i.e.*, all samples from each participant were analyzed in the same run, except for analysis of calcium, creatinine, and phosphate, which were analyzed within two hours after collection. A second void morning urine sample was collected at home. Urine samples were collected under fasting conditions or before any consumption of calcium rich foods. Plasma 25-hydroxyvitamin D (25OHD) concentrations were measured by isotope dilution liquid chromatography–tandem mass spectrometry (LC-MS/MS) by a method adapted from Maunsell *et al.* [23,24]. The method separately quantifies 25OHD_2_ and total 25OHD_3_ (including the 3-epimer). The total 25OHD concentration was calculated and used for further analyses. Calibrators traceable to NIST SRM 972 (Chromsystems, DE) were used. The inter-assay CV was <10%, at plasma concentrations of 23.4 nmol/L (25OHD_2_) and 24.8 nmol/L (25OHD_3_). We determined plasma 1,25-dihydroxyvitamin D (1,25(OH)_2_D) concentrations by a radioimmunoassay (Gamma-B 1,25-Dihydroxy Vitamin D, Immunodiagnostic Systems (IDS) Ltd., Boldon, England). The inter- and intra-assay CV was 9.0% and 8.0%, respectively, at 220 pmol/L.

Vitamin D binding protein concentration was determined by ELISA (R & D Systems, Abingdon, UK) with both an inter- and intra-assay CV < 6%. Assay performance was monitored using kit and in-house controls and under strict standardization according to ISO 9001:2000.

The free fraction of 25OHD and 1,25(OH)_2_D were calculated as the free 25OHD index (FI-25OHD) and the free 1,25(OH)_2_D index (FI-1,25(OH)_2_D) using the molar ratio of 25OHD and 1,25(OH)_2_D to VDBP [11].

We determined plasma and urinary concentrations of calcium and creatinine (Cr) by standard laboratory methods and calculated the albumin adjusted calcium concentration according to the formula: plasma calcium, adjusted [mmol/L] = plasma calcium, total [mmol/L] + 0.00086 × (650-plasma albumin µmol/L) [22].

Calcitonin was measured by a radioimmunoassay as described by Schifter [25]. The plasma concentrations of intact parathyroid hormone (PTH) and osteocalcin were measured with electro-chemiluminescence immunoassays using an automated instrument (Cobas 601e, Roche Diagnostics, GmbH, Mannheim, Germany). We measured plasma bone specific alkaline phosphatase concentrations by an immunoassay (METRA BAP EIA kit, Quidel Corporation, San Diego, CA, USA). The renal excretion of cross-linked *N*-terminal telopeptide of type 1 collagen (NTx) was quantified by ELISA using an automated instrument (Vitros ECI, Ortho Clinical Diagnostics, Amersham, UK). Results were expressed relative to creatinine (Cr) excretion (NTx/Cr), as nmol of bone collagen equivalents (nMmol BCE) per mmol of creatinine. The CV was 9.6% at 41.5 nmol BCE/mmol Cr.

We measured bone mineral density (BMD) of the whole body, the lumbar spine, and total hip. Total body fat and lean mass were measured. All DXA scans were performed using a Hologic Discovery scanner (Hologic, Waltham, MA, USA). We assessed long-term stability through daily scans of an anthropometrical phantom. Precision error for BMD was 1% at the lumbar spine and 2% at the total hip. 

### 2.3. Statistics

The majority of the data were non-normally distributed, therefore descriptive statistics are reported as medians with the 25 and 75-percentile (p25; p75), unless stated otherwise. We explored the differences between groups using chi-square tests for categorical variables and a Mann-Whitney *U*-test for continuous variables. Spearman’s rho correlation was used to calculate the magnitude and direction of the correlations between measured variables.

Vitamin D status was described according to plasma 25OHD concentrations categorized into three groups: 25OHD < 50 nmol/L; 25OHD between 50.1 and 75 nmol/L, 25OHD > 75.1 nmol/L [26].

All statistical analyses were performed using the Statistical Package for Social Sciences (SPSS 17, Chicago, IL, USA) for Windows. *P*-values below 0.05 were considered statistically significant.

## 3. Results

Table 1 shows characteristics of the 75 included women. Anthropometric-, diet-, and lifestyle-characteristics did not differ between women using hormonal contraceptives (HC) (*n =* 52) and non-users (*n =* 23), except that daily calcium intake was slightly higher in users- compared with non-users of HC (*p =* 0.02).

**Table 1:** Characteristic of the 75 studied women stratified by use of hormonal contraceptives. Median with interquartile (p25; p75) ranges unless otherwise indicated.

	All, *n =* 75	Users of hormonal contraceptive (*n =* 52)	Non-users of hormonal contraceptives (*n =* 23)	*p* value ^1^
Age, mean	29 (27; 32)	29 (27; 32)	29 (26; 33)	0.81
Weight, kg	67 (60; 77)	69 (63; 77)	63 (57; 80)	0.12
Height, cm	168 (163; 172)	167 (162; 172)	169 (163; 172)	0.61
BMI	24 (22; 27)	25 (23; 27)	23 (20; 27)	0.12
Total calcium intake, mg/day	800 (660; 975)	850 (700; 1000)	700 (500; 853)	*0.02*
Use of vitamin D supplements, *n* (%) Vitamin D intake from supplements (µg/day)	24 (32) 5 (5; 9)	17 (33) 5 (5; 10)	7 (30) 5 (5; 5)	1.00
Smoking, *n* (%)	11 (15)	7 (14)	4 (17)	0.71

^1^ Independent-Samples Mann-Whitney U Test.

Of the 52 HC users, 44 used oral HC, six used intrauterine hormonal device, one used sub-dermal contraceptive implant, and one used a vaginal ring. No differences in biochemical markers were seen between the 44 using oral HC and the six using intrauterine hormonal device (data not shown).

Physical activity, time spend outdoor, and the time of the day for blood sampling did not differ between the HC users and no-users of HC (data not shown). The number of women with a BMI above 25 was not different between users- and non-HC users. BMD at whole body, lumbar spine, and total hip, as well as fat and lean mass did not differ between the HC users and no-users of HC (data not shown). However, given the sample size of 23 non-HC users and 52 users our statistical power to detect a 5% difference between groups in lumbar spine BMD (2α = 0.05 and β = 0.20) was only approximately 60%.

**Figure 1:**
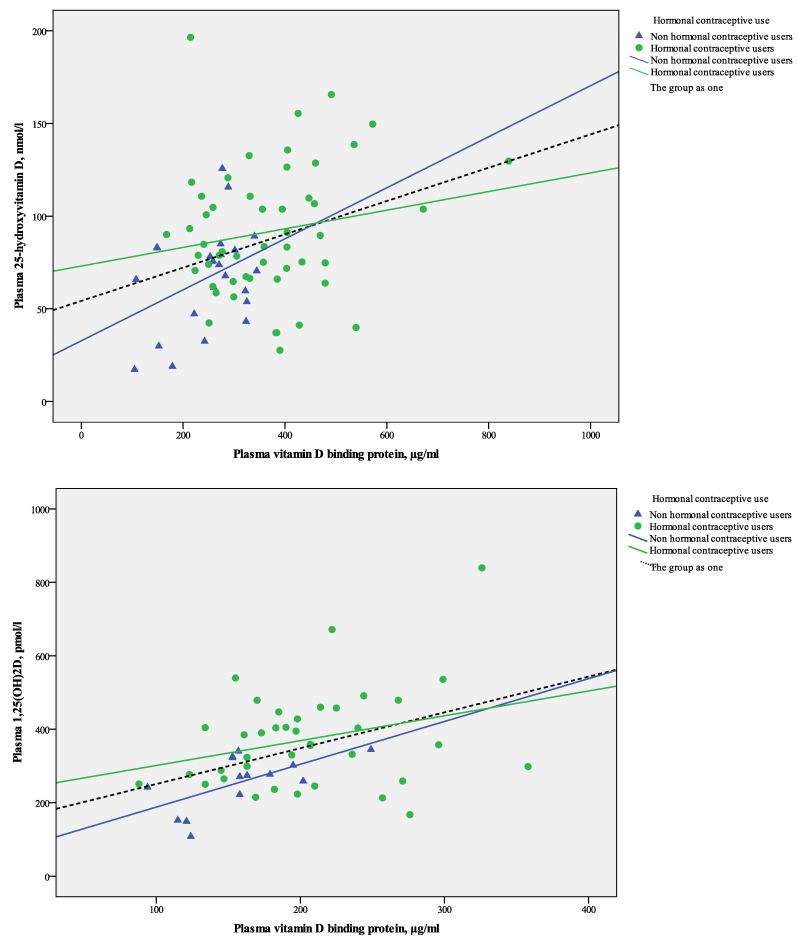
(**A**) Scatter plot of linear relations between plasma 25OHD and VDBP in 75 healthy Caucasian women stratified by use of hormonal contraceptives; (**B**) Scatter plot of linear relations between plasma 1,25OH_2_D and VDBP in 75 healthy Caucasian women stratified by use of hormonal contraceptives.

When all data were pooled, the plasma concentrations of 25OHD was significantly and positively correlated with VDBP (*r*_s_ = 0.26, *p =* 0.03) (Figure 1A) and further with 1,25(OH)_2_D (*r*_s_ = 0.43, *p <* 0.01).

Plasma 1,25(OH)_2_D was significantly and positively correlated with VDBP concentrations (*r*_s_ = 0.39, *p <* 0.01) when data for all women (HC users and non users) were pooled (Figure 1B).

### 3.1. The Effect of Use of Hormonal Contraceptives on P-25OHD, P-1,25OH2D, and P-VDBP Concentrations

Table 2 details the biochemical indices measured as stratified by whether studied women used HC.

**Table 2:** Biochemical characteristics as stratified by use of hormonal contraceptives. Data are presented as Median with interquartile (p25; p75) ranges unless otherwise indicated.

	All, *n =* 75	Users of hormonal contraceptive users (*n =* 52)	Non-users of hormonal contraceptives (*n =* 23)	*p* value ^1^
Plasma vitamin D binding protein, µg/mL	305 (251; 404)	358 (260; 432)	271 (179; 302)	<0.001
Plasma 25-hydroxyvitamin D, nmol/L	79 (64; 104)	84 (67; 111)	70 (47; 83)	0.01
Free index 25-hydroxyvitamin D (×10^−3^)	14 (10; 19)	14 (10; 19)	15 (10; 17)	0.84
Plasma 1,25-dihydroxyvitamin D, pmol/L	185 (156; 224)	198 (163; 241)	158 (123; 183)	0.01
Free index 1,25-dihydroxyvitamin D (×10^−6^)	31 (26; 41)	29 (25; 41)	36 (27; 43)	0.10
Vitamin D status, *N* (%)				
<50 nmol/L	12 (16)	6 (12)	6 (50)	
50–75 nmol/L	19 (25)	12 (23)	7 (37)	0.15 ^2^
>75 nmol/L	44 (59)	34 (65)	10 (23)	
Plasma PTH, pmol/L	3.6 (2.9; 4.6)	3.3 (2.5; 4.3)	3.8 (3.4; 4.6)	0.36
Plasma calcium total, albumin adjusted, mmol/L	2.45 (2.42; 2.52)	2.46 (2.43; 2.51)	2.45 (2.40; 2.53)	0.94
Plasma phosphate, mmol/L	1.00 (0.93; 1.12)	0.97 (0.89; 1.09)	1.03 (0.95; 1.21)	0.05
Plasma creatinine, µmol/L	64 (57; 72)	65 (57; 73)	61 (58; 69)	0.22
Plasma calcitonin, pmol/L	10 (9; 12)	10 (9; 12)	9 (8; 11)	0.21
Plasma bone specific alkaline phosphatase, U/L	17.9 (14.8; 23.0)	16.5 (14.6; 21.0)	21.1 (14.8; 23.8)	0.22
Plasma osteocalcin, µg/L	26.9 (19.3; 30.9)	25.9 (19.0; 29.6)	29.6 (20.6; 39.2)	0.07
Urine NTx/creatinine ratio (mmol/mmol)	42.6 (30.9; 53.0)	39.3 (29.5; 50.8)	48.7 (38.8; 57.8)	0.11
Urine calcium/creatinine-ratio (mmol/mmol)	0.2 (0.1; 0.4)	0.2 (0.1; 0.4)	0.3 (0.1; 0.4)	0.52

^1^ Independent-Samples Mann-Whitney U Test; ^2^ Chi-Square Tests.

Compared with the non-users, HC users has a significantly higher plasma concentrations of 25OHD, 1,25(OH)_2_D, and VDBP (*p <* 0.01). The median plasma concentrations of 25OHD, 1,25(OH)_2_D, and VDBP were respectively 16%, 13%, and 25%, higher in users compared to non-users of HC. However, FI-25OHD and FI-1,25(OH)_2_D did not differ between groups.

Adjustment for between group differences in body weight did not change results.

The prevalence of a 25OHD concentration below 50 nmol/L was equal between groups, whereas there were three times as many users with a 25OHD concentration above 75.1 nmol/L as non-users of HC (Table 2).

The correlation between the plasma concentration of 25OHD and 1,25(OH)_2_D was near significant in both users of HC (*r*_s_ = 0.31, *p =* 0.06) and in non-users of HC (*r* = 0.49, *p =* 0.07). 

However, 25OHD and VDBP concentrations were not significantly correlated when groups were analyzed separately (in HC users: *r*_s_ = 0.15, *p =* 0.29 and in non-users: *r*_s_ = 0.29, *p =* 0.18). Plasma 1,25(OH)_2_D tended to be positively correlated with VDBP in non-users of HC (*r*_s_ = 0.52, *p =* 0.06), but not in HC users (*r*_s_ = 0.21, *p =* 0.21) (Figure 1B). 

### 3.2. The Effect of Use of Hormonal Contraceptives on Calcium Homeostasis and Bone Turnover

As shown in Table 2, plasma concentrations of phosphate and osteocalcin were borderline significant lower in users-compared with non-users of HC; whereas no other measured indices differed between groups. 

## 4. Discussion

We have studied a group of healthy young Danish women among whom 52 used HC and 23 did not. Our analyses showed significantly higher plasma concentrations of 25OHD, 1,25(OH)_2_D and VDBP in users compared with non-users of HC, FI-25OHD and FI-1,25OH_2_D were however not different between groups.

Our findings of increased VDBP concentrations in users of HC agrees with the findings in postmenopausal women receiving postmenopausal hormone substitution. In an earlier study from our group, initiation of postmenopausal oestrogen therapy caused a significant 8% increase in VDBP concentrations [19]. Similar results have been reported in pregnancy, during which an increase in VDBP concentration is observed [10,18].

Plasma 1,25(OH)_2_D is known to suppress the secretion of PTH, stimulate the synthesis of osteocalcin and enhance intestinal absorption of calcium and phosphate [27,28]. The latter may be reflected in an increase renal excretion of these minerals [27]. Despite a significant increase in plasma 1,25(OH)_2_D our data did not show any significant effect of HC on indices of calcium and phosphate homeostasis or bone metabolism. These findings may support the notion of the free hormone hypothesis, *i.e.*, that only the free fraction of the hormone has biological effects [29].

We assume based on our results and previous reports [5,10,15,18,19] that the estrogen component of HC may increase VDBP synthesis or decrease its catabolism. The concomitant increase in the total plasma 1,25(OH)_2_D concentration may mirror a compensatory adjustment to maintain an unaltered concentration of the free fraction [10,16,18].

VDBP binding protects vitamin D metabolites from hydroxylase-mediated catabolism; an increase in VDBP may therefore reduce further metabolism of vitamin D metabolites, increasing their half-life. An alternative explanation is that, in parallel with the up regulation of the 1,25(OH)_2_D concentration, the total 25OHD concentration is unregulated via unknown mechanisms, to maintain the free concentration of 25OHD, available to tissues. This may potentially explain the higher plasma concentration of the largely unregulated plasma concentration of 25OHD in HC users, however, this needs further investigation.

An important limitation of our study is the relative small sample size and the fact that women were healthy and all had plasma 25OHD concentrations over 25 nmol/L. This may have limited our ability to detect further potential effects of HC on calcium homeostasis and bone metabolism through variations in VDBP, 25OHD, and 1,25(OH)_2_D in vitamin D deficiency (25OHD < 25 nmol/L). Further studies should therefore aim to investigate effects of HC in women with vitamin D deficiency. Moreover, investigations in larger groups are needed to assess the effects of HC on vitamin D metabolites and its effect on muscle and bone, as well as other health outcomes.

## 5. Conclusions

In conclusion, use of HC is associated with an elevated plasma concentration of VDBP and concomitant higher plasma 25OHD and 1,25(OH)_2_D. The free-indices of these vitamin D metabolites are however similar to non-users of HC. The point of emphasis: the use of HC should be considered in the interpretation of 25OHD and 1,25(OH)_2_D vitamin D concentrations in women. Further studies should aim to clarify whether also in women with a low vitamin D supply, an HC induced increase in VDBP is accompanied by an increase in plasma 25OHD to maintain the free 25OHD level. Further research is also required to assess whether the free 25OHD index is a better marker of 25OHD tissue availability and has a higher correlation with indices of calcium homeostasis and bone metabolism than total 25OHD levels.
